# Experimental Investigation and Prediction of Mechanical Properties of Carbonate Rocks Under Uniaxial and Triaxial Compressions

**DOI:** 10.3390/ma18061211

**Published:** 2025-03-08

**Authors:** Esraa Alomari, Kam Ng, Lokendra Khatri

**Affiliations:** Department of Civil and Architectural Engineering and Construction Management, University of Wyoming, Laramie, WY 82071, USA; esraa.alomari@wsp.com (E.A.); lokendra.khatri320@gmail.com (L.K.)

**Keywords:** carbonate rocks, compressive strength, confining pressure, porosity, Young’s modulus, wing crack model

## Abstract

Compressive strength and Young’s modulus are key design parameters in rock engineering, essential for understanding the mechanical behavior of carbonate rocks. Understanding the mechanical behavior of carbonate rocks under varying load conditions is crucial for geotechnical stability analysis. In this paper, empirical relationships are developed to predict the mechanical properties of carbonate rocks. A series of uniaxial and triaxial compression experiments were conducted on carbonate rocks including limestone, dolostone, and granite from Wyoming. In addition, experimental data on different carbonate rocks from the literature are compiled and integrated into this study to evaluate the goodness of fit of our proposed empirical relationships in the prediction of compressive strength and Young’s modulus of carbonate rocks. Regression analysis was used to develop predictive models for the uniaxial compressive strength (UCS), Young’s modulus (E), and triaxial compressive strength ($\sigma_{1}$) incorporating parameters such as the porosity (*n*) and confining pressure ($\sigma_{3}$). The results indicated that the *UCS* and Young’s modulus showed a power relationship with porosity (*n*), whereas the $\sigma_{1}$ showed a linear relationship with *n* and $\sigma_{3}$. Furthermore, an analytical model expanded from the wing crack model was applied to predict the $\sigma_{1}$ of limestone based on the coefficient of friction, the initial level of damage, the initial flaw size, and the fracture toughness of the rock. The model showed a good predictability of the $\sigma_{1}$ with a mean bias (i.e., the ratio of the measured to the predicted strength) of 1.07, indicating its reliability in accurately predicting the rock strength. This predictability is crucial for making informed engineering decisions, design optimization, and improving safety protocols in practical applications such as structural analysis and manufacturing processes.

## 1. Introduction

Carbonate rocks are widely considered to be one of the best reservoir rocks for oil exploration [1]. Understanding the mechanical behavior of carbonate rocks has broad implications for reservoirs and earthquake engineering [2]. In the context of reservoirs, carbonate rocks such as limestone and dolomite are often the primary reservoir rocks for oil and gas, whereas, from an earthquake engineering perspective, carbonate rocks also play a significant role in fault zones, where their brittleness and low porosity can lead to rapid stress release during seismic events. In rock engineering, mechanical properties such as uniaxial compressive strength (*UCS*) and Young’s modulus (*E*) are commonly used parameters, as they are essential for intact rock classification and failure criteria determination [3]. However, due to their inherent instability in their texture and mineral composition, as well as their high reactivity with water and temperature changes, carbonate rocks are highly susceptible to weathering [4]. Consequently, obtaining high-quality core samples with sufficient quantities for laboratory testing can be challenging; therefore, developing equations for predicting the mechanical properties of carbonate rocks is of great interest.

Various studies have reported relationships between *UCS* and porosity (*n*) for carbonate rocks [5,6,7,8,9], including dolomite [5,10], and limestone [5,11]. Generally, these relationships indicate that *UCS* decreases as the porosity increases. It is well established that pores act as stress concentrators, thereby influencing the overall strength of the rock, particularly when pores are oriented along stress planes where they can act as planes of weakness or filled with fluids where they can increase pore pressure and reduce effective stress [12]. However, most of these empirical relationships are specific to certain rock formations and may not be applicable to other rock types. Also, a lot of these relationships define an upper limit for porosity. For instance, the *UCS* of different carbonate rocks such as Brecciated Limestone and Jurassic Dolomite follows a logarithmic relationship with porosity values less than 30% [8]. Similarly, the *UCS* of carbonate rocks collected from the Korobcheyev deposit in Russia shows an exponential relationship with porosity values ranging from 5% and 20% [5]. Therefore, further investigation to explore a broader range of formations and porosities is required, which is presented in this current study.

Fereidooni et al. [9] conducted a study to estimate the *UCS* and *E* of intact carbonate rocks using non-destructive testing and ensemble learning models. To enhance the accuracy of *UCS* estimation, they implemented stacking ensemble learning models, integrating Multi-Layer Perception (MLP), Random Forest (RF), Support Vector Regressor (SVR), and Extreme Gradient Boosting (XGBoost). The developed models demonstrated high predictive performance, with a coefficient of determination of 0.909 for *UCS* estimation, indicating strong reliability. However, machine learning models can sometimes capture noise or biases present in the training set, which may affect their generalizability to broader datasets or different geological formations.

Young’s modulus (*E*) of carbonate rocks including limestone and dolomite collected from the Ghawar field located in the Eastern Province of Saudi Arabia is related to porosities of less than 30% [13]. Similarly, another study conducted by Asef et al. [14] reported that *E* is related to *n* less than 30% for carbonate rocks collected from different locations in Iran. However, these relationships yield satisfactory results under a specific porosity range and do not work well otherwise, as shown later in this study. Most of the relationships developed for carbonate rocks are derived for a lower porosity range (i.e., less than 30%); therefore, for a higher range of porosity (i.e., higher than 30%), the predictive accuracy of these relationships decreases. Hence, the relationships proposed in this paper are designed to apply to carbonate rock types with a broader range of porosities.

A study by Bakun-Mazor et al. [15] explored the use of hyperspectral remote sensing to non-destructively predict the mechanical and physical properties of carbonate rocks. The study included testing 150 cylindrical samples for *UCS*, density, porosity, and water absorption. By analyzing the spectral signature of these samples, they developed models that accurately predict these properties. The study highlights the potential of spectroscopy as a rapid and effective tool for assessing rock mechanical characteristics. However, the accuracy of their models relies heavily on the spectral signature of rocks, which varies with the mineral composition, surface conditions, and environmental factors, making the models less generalizable across different geological conditions. Also, spectroscopy requires specialized equipment and calibration, which may not be readily available in many field or industrial settings.

Hatzor and Palchik [16] developed an empirical model that accounts for the effect of microstructure on compressive strength ($\sigma_{1}$) of Aminadav dolomite. According to the model, $\sigma_{1}$ increases with the inverse square root of mean grain size and exhibits a non-linear increase with confining pressure ($\sigma_{3}$). However, the model includes three empirical parameters (i.e., *a*, *b*, and *c*) that are estimated by fitting experimental data specifically for the Aminadav dolomite, limiting its applicability to carbonate rocks in general.

To address the limitations mentioned, several empirical relationships have been proposed to estimate the rock strength and stiffness using measurable parameters such as porosity (*n*). These relationships are often the only viable method for estimating the mechanical properties of carbonate rocks in the absence of testable core samples. The novelty of this study lies in the development of empirical models that can predict the mechanical properties of carbonate rocks over a broader range of porosities, including those exceeding 30%. Unlike previous models, which primarily focus on lower porosity ranges, the proposed model enhances predictive accuracy for rocks with higher porosity. Additionally, this study incorporates both porosity and confining pressure (*σ*_3_) to assess compressive strength (*σ*_1_), offering a more comprehensive approach. Furthermore, the expanded wing crack model applied here introduces a new method for predicting triaxial compressive strength by considering secondary crack interactions and fracture mechanics, providing more reliable estimates for carbonate rock failure under complex stress conditions.

A series of uniaxial and triaxial compression tests were performed by the authors on different carbonate rocks including limestone, granite, and dolomite. We investigated the effect of the *n* on the *UCS* and *E* in addition to the study of the combined effect of *n* and $\sigma_{3}$ on $\sigma_{1}$. Triaxial compression data for tested rocks from Wyoming and experimental data from published literature were analyzed to develop empirical equations. Regression analysis was adopted to develop relationships to estimate the *UCS*, *E*, and $\sigma_{1}$ of carbonate rocks with relative confidence. The goodness of fit for our proposed empirical equations is evaluated by comparing the predicted with the measured *UCS*, *E*, and $\sigma_{1}$ values. In addition, the comparison of our proposed equations and other relationships from the literature is conducted. Additionally, an expanded wing crack model proposed by the authors [17] and derived from an original wing crack model [18] was applied to predict the triaxial compression strength of limestone, and the predictability of this model was evaluated by comparing the predicted strength with the measured strength. The expanded wing crack model aims to determine the normalized critical crack length based on fracture mechanics applied to secondary cracks emanating from pre-existing flaws and interacting to eventually cause failure [17].

## 2. Materials and Methods

### 2.1. Uniaxial Compressive Strength (UCS)

#### 2.1.1. Sample Preparation and Test Equipment

The study area is located in Wyoming, USA, and the collected samples from various formations are summarized in Table 1. The carbonate rock samples from Wyoming were specifically selected due to their wide availability and diversity in mineral composition, which make them ideal for understanding the mechanical behavior of carbonate rocks in various conditions. Also, Wyoming is known for its carbonate rock formations, including a range of limestone and dolomite varieties, with differing porosity and mechanical properties, which provides a comprehensive dataset for developing and testing empirical models [19]. Three limestone samples of the Permian, Mississippian, and Devonian geological ages were collected from the surface of Toms Pit (Washakie County, Wyoming). In addition, two granite samples from the Precambrian and Proterozoic ages as well as one Ordovician dolostone were collected. Each rock specimen was prepared with 25 mm diameter by 50 mm height from rock blocks collected from the field, and both ends of the rock specimens were trimmed and polished. Due to the difficulty in preparing samples to meet the required geometry and structural integrity standards, a significant number of samples were lost, thereby, only representative carbonate rock formations were included for testing. All specimens were tested at room temperature. The porosity (*n*) of each specimen is determined using the gravimetric method where the specific gravity is determined according to the AASHTO T-100 standard test method [20]. The *n* is calculated as

(1) $$n\;\left(\%\right)=\frac{1-dry\;bulk\;density}{specific\;gravity}\times 100$$

**Figure 1:**
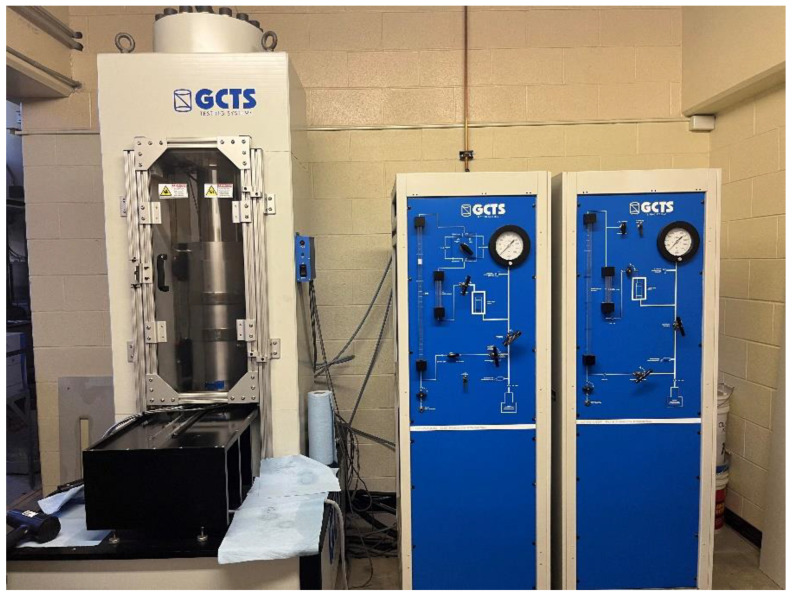
Testing equipment GCTS Rapid Triaxial Rock (RTR-1500) for triaxial compression testing.

Uniaxial and triaxial compression tests were conducted using a servo-controlled testing equipment GCTS Rapid Triaxial Rock (RTR-1500, GCTS, Phoenix, AZ, USA) at the University of Wyoming, USA as shown in Figure 1. The system is equipped with a load frame stiffness of 1.75 MN/mm and includes a fully integrated SCON-2000 digital signal controller (GCTS, Phoenix, AZ, USA) and CATS-TRX-ROCKS software (GCTS, Phoenix, AZ, USA). The axial load actuator has a maximum capacity of 1500 kN and a maximum confining pressure capacity of 140 MPa achieved by filling the chamber with oil. A heat shrink membrane is used to protect the rock specimen from the oil during testing. For strain measurement, two axial and one radial Linear Variable Differential Transformers (LVDTs) were attached to the specimen.

#### 2.1.2. Uniaxial Compression Testing

A total of five carbonate specimens, representing different formations and porosities, are tested under a uniaxial compression (UC) condition at room temperature. The tested samples include two limestone samples, two granite samples, and one dolostone sample, all of which were collected from the surface. A summary of geological, physical, and mechanical information of the tested samples is given in Table 1. The tested samples have *n* values ranging from 0.81 to 12.41% and *UCS* values ranging from 11.33 to 87.97 MPa. The *E* of the tested samples ranges from 9.83 to 34.41 GPa.

#### 2.1.3. UC Data of Carbonate Rocks from the Literature

Experimental data compiled from the literature are utilized to better understand the mechanical behavior of carbonate rocks from various regions around the world. Thirty-three distinct carbonate formations, including limestone, dolomite, dolostone, chalk, marble, and gypsum, were tested under uniaxial compression UC, and their properties are summarized in Table 2. The tested samples have *n* values ranging from 0.40 to 50.10%, *UCS* values ranging from 0.63 to 203.00 MPa, and *E* values ranging from 0.47 to 80 GPa.

### 2.2. Triaxial Compressive Strength ($\sigma_{1}$)

#### 2.2.1. Conventional Triaxial Compression Testing

A series of triaxial tests were performed on intact specimens to assess the mechanical properties of carbonate rocks and to develop new predictive equations. Experimental data from Wyoming, along with additional data collected from the literature, were used to analyze the mechanical behavior and failure mechanism of carbonate rocks. The experimental data of carbonate rocks from Wyoming are summarized in Table 3.

During each triaxial experiment, the cell wall is filled with oil, and the desired confining pressure is applied to the rock specimen. An initial seating pressure of 0.345 MPa is applied before the shearing stage begins. The rock is then subjected to axial shearing using a controlled axial strain system. The test is terminated once the rock fails [21]. The triaxial compression test setup was specifically designed to simulate the confining pressures that carbonate rocks experience in natural geological settings. In reservoir environments, for example, rocks are subjected to high confining pressures due to the overburden and surrounding geological formations, which influence their mechanical behavior [22]. Similarly, in fault zones, carbonate rocks can experience varying levels of confining pressure, depending on the depth and tectonic activity [23]. By applying confining pressures in the laboratory, the triaxial compression test mimics these real-world stress conditions, allowing for a more accurate understanding of the mechanical properties (e.g., strength and stiffness) of the rocks under realistic field conditions. The limitation of triaxial compression test is limited to its ability to simulate the actual polyaxial stress condition in deep reservoirs.

**Figure 2:**
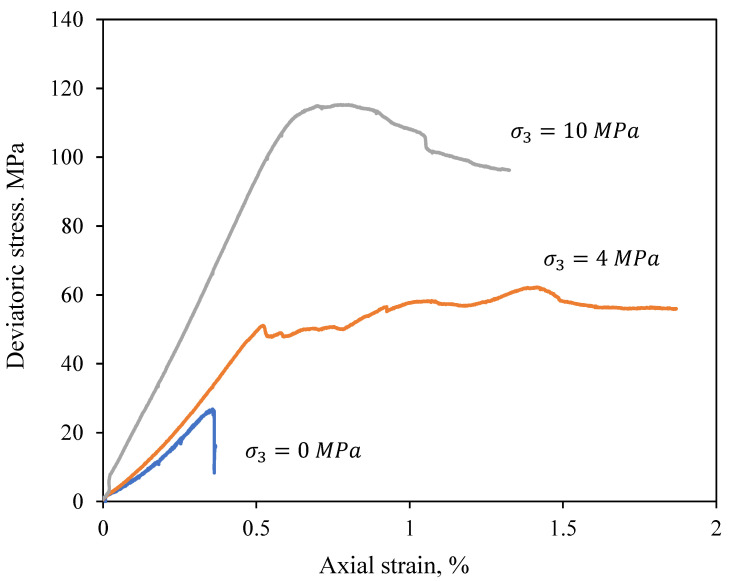
Three deviatoric stress–strain curves of Sherman granite specimens under confining pressures of 0, 4, and 10 MPa.

The deviatoric stress–strain curves of the Sherman granite sample under different confining pressures are demonstrated in Figure 2 as an example. The deviatoric stress is calculated as $\sigma_{1}-\sigma_{3}$, where $\sigma_{1}$ and $\sigma_{3}$ are the major and minor principal stresses, respectively. A non-linear deformation of the stress–strain curves was observed at the beginning of loading, particularly under lower confinement, due to the closure of the initial pores and flaws [24]. As the confining pressure increases, the initial deformation is reduced. The compressive strength is represented by the peak stress value, followed by the post-peak stage.

**Table 2:** Summary of UC test results of carbonate rocks from literature.

Rock Formation	Country of Origin	*n,* %	*UCS*, MPa	*E*, GPa	Reference
Madison Limestone	Wyoming, USA	6.50–8.00	26.65–76.65	NA	[25]
Yarka Limestone	Judea group in Israel	15.70–17.90	38.70–71.00	6.20–8.40	[26]
Devonian Limestone	Turkey	1.14–4.12	74.20–138.10	16.68–46.23	[27]
Savonnieres Limestone	Jordan	30.60–36.10	11.20–17.00	NA	[28]
Brauvilliers Limestone		27.00–33.70	11.90–23.20	NA	
Anstrude Limestone		18.10–21.90	41.10–58.10	NA	
Kirechane Limestone	Turkey	4.90–33.90	7.32–24.06	NA	[29]
Miocene Limestone	Budapest, Hungary	11.40–52.20	0.63–27.6	0.47–10.30	[30]
Akveren Limestone	Turkey	2.20–2.60	28.00–33.00	49.00–58.69	[31]
Akiyoshi Limestone	Japan	0.50–0.90	75.00–101.00	NA	[32]
Asmari Limestone	Iran	2.04–7.21	50.40–84.20	NA	[33]
Indiana Limestone	Canada	14.80	61.00	NA	[34]
Reef Limestone	South China sea	2.30	42.00	NA	[35]
Triassic Dolostone	Italy	0.76–4.70	47.29–112.00	2.38–18.80	[36]
Brecciated Dolostone		1.40–4.60	15.18–109.65	2.67–18.09	
Weathered Limestone	Netherlands	8.10	39.00	37.00	[37]
Artificial fine-grained Gypsum rock		35.00	8.00	2.00	
Medium grained Calcarenite		50.10	6.00	NA	
Detrital Limestone		19.80	22.00	9.00	
Fine-grained Limestone		37.90	31.00	12.00	
		15.70	57.00	24.00	
Weathered Dolomite		12.70	39.00	38.00	
Weathered Limestone		8.10	39.00	37.00	
Fine-grained Dolomite		10.70	67.00	32.00	
Fine-grained Marble		0.40	94.00	49.00	
Fresh micritic fine grained Limestone		5.40	101.00	26.00	
Fine-grained micritic Limestone		4.90	74.00	52.00	
Coarse crystalline Limestone		1.10	85.00	59.00	
Medium grained Limestone		3.80	174.00	59.00	
		1.00	176.00	78.00	
		0.60	159.00	76.00	
		0.70	203.00	80.00	
Fine-grained Limestone		0.50	163.00	69.00	
Crystalline Limestone, china		0.80	186.00	70.00	
Morawica	Poland	3.40	120.00	58.00	[38]
Tonnerre Limestone	France	13.70	72.40	19.30	
Chauvigny Limestone		17.40	42.00	16.30	
Lavoux Limestone		21.80	30.40	13.80	
Louny Gauze	Poland	26.00	58.00	9.20	
Lixhe Chalk	Belgium	42.00	7.70	3.80	
Karaman Travertine	Turkey	2.15–13.27	45.40–112.30	NA	[39]
Danian Chalk	Texas, USA	43.05	11.00	NA	[40]
Austin Chalk		25.75	25.00	NA	
Devonian Limestone	Texas, USA	2.30	78.45	NA	[41]
Fusselman Limestone		3.00	39.23	NA	
Wolfcamp Limestone	New Mexico, USA	4.20	110.82	NA	
Soignies Limestone	Belgium	0.40	170.00	NA	[42]
		0.40	139.00	13.20	
Moca Limestone		8.00	79.00	NA	
Sorcy Limestone		30.00	47.00	NA	

*n*—Porosity (%); *UCS*—unconfined compressive strength (MPa); and *E*—Young’s modulus (GPa).

**Table 3:** Summary of the triaxial test results of carbonate rock formations in Wyoming.

Sample	Specimen ID	Rock Type	Formation	Geological Age	*n*, %	$\sigma_{3}$, MPa	$\sigma_{1}$, MPa
25	25 a	Limestone	Madison	Mississippian	7.93	1	38.61
	25 b				2.60	4	90.05
	25 c				3.23	10	31.68
27	27 b	Limestone	Goose Egg	Permian	11.96	1	67.03
	27 c				12.18	2	63.00
	27 d				12.23	8	119.54
47	47 b	Limestone	Jefferson	Devonian	1.55	4	52.77
	47 c				1.90	10	146.74
28	28 b	Granite	NA	Precambrian	0.69	1	34.67
	28 c				0.72	6	154.61
	28 d				0.98	10	200.22
55	55 a	Granite	Sherman	Proterozoic	4.85	4	66.26
	55 b				4.38	10	125.28
45	45 b	Dolostone	Big Horn	Ordovician	8.27	4	79.56
	45 c				8.09	10	38.55

*n*—Porosity in percentage; $\sigma_{3}$—confining pressure in MPa; $\sigma_{1}$—peak compressive strength in MPa; and NA—not available. a—d are the specimen identification of the same sample

#### 2.2.2. Triaxial Data of Carbonate Rocks from the Literature

Triaxial experimental data of forty-one different carbonate formations were collected from the literature to investigate the combined effect of porosity and confining pressure on the compressive strength. The rocks tested included limestone, grainstone, marble, dolomite, and gypsum, and they were tested under confining pressures ranging from 0.1 to 800 MPa. These formations, which exhibit a broad range of porosities from 0.02 to 37% and a wide variation in compressive strength (i.e., $\sigma_{1}$ ranging from 20 to 1760 MPa) are summarized in Table 4.

## 3. Results

### 3.1. Results of Uniaxial Compressive Strength (UCS)

#### Relationship Between UCS and Porosity

Uniaxial compressive strength (*UCS*) is one of the most frequently measured parameters in rock engineering [67]. Understanding the relationship between mechanical and physical properties is essential in geotechnical applications such as hydraulic fracturing [68]. The dataset was randomly split into training and testing sets [69] using RStudio software version 2022.02.2 [70]. The training dataset was used to develop empirical equations, while the testing dataset was used to assess the predictive accuracy of the proposed equations. The data were split with 70% allocated to the training set and 30% to the testing set.

**Figure 3:**
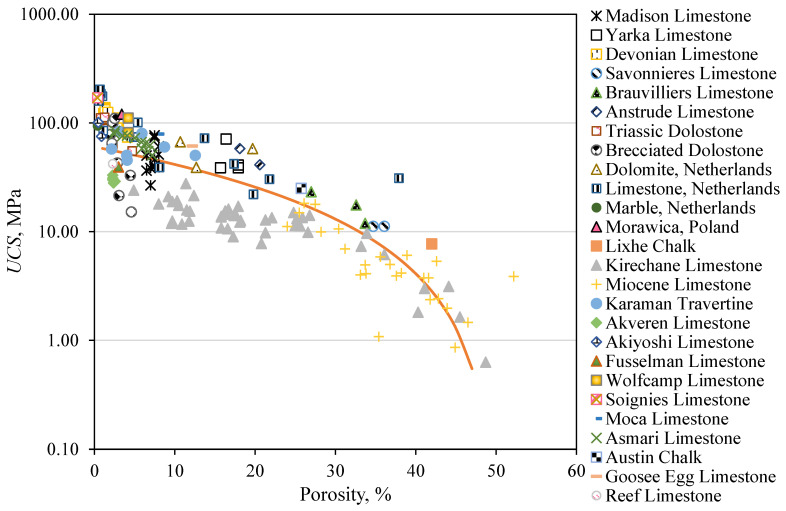
The negative relationship between the rock *UCS* and porosity, indicating how increased rock porosity can lead to strength reduction.

A power relationship, as shown in Equation (2), was developed to describe the relationship between the *UCS* and porosity (*n*), based on the scatterplot of the response variable *UCS* versus the predictor variable *n.* This equation was derived based on the training dataset that contains 178 data points of different rock formations (i.e., four data points are from Wyoming and the remaining 174 data points are from the literature) as shown in Figure 3. The scatter observed in the *UCS*—porosity plot can be attributed to several factors influencing the rock’s mechanical and physical properties such as variations in mineral composition, as different minerals exhibit varying degrees of strength and porosity [71]. Additionally, grain size and the degree of cementation can introduce further variability [72]. Heterogeneities such as fractures or varying degrees of weathering within the rock may also contribute to the scatter by influencing local stress distribution [73]. These factors highlight the complexity of the relationship between *UCS* and porosity. The results indicate that even a small increase in *n* (~1–5%) leads to a 13% decrease in *UCS*. The high sensitivity of *UCS* to changes in *n* is reflected by the exponent *β* of 1.67. Equation (2) accounts for two asymptotes; the ${UCS}_{o}$ at zero porosity which is crucial for understanding the strength of fully intact rock without any pore space and the vanishing *UCS* (i.e., as the porosity approaches the maximum value $n^{*}$) (see also Ng and Santamarina [11]):

(2) $$\hat{UCS}\;\left(MPa\right)={UCS}_{o}\;{\left(1-\frac{n}{n^{*}}\right)}^{\beta }=60.251\;{\left(1-0.02\;n\right)}^{1.67}$$

In Equation (2), $n^{*}$ is assumed to equal 0.5 for carbonate rocks, and the experimental fitted data show that the average *UCS* at zero porosity ${UCS}_{o}=60.251\;MPa.$ The equation demonstrates the negative effect of porosity (*n*) on *UCS*. This is explained by the fact that the porosity represents the void spaces in the rock, which are considered weak points within the rock matrix. Consequently, the more porous the rock is, the more voids it has, which reduces the strength of the rock skeleton [74]. For instance, the *UCS* of Kirechane Limestone (given in Table 2) decreases from 22.69 to 9.63 MPa or 58% when the *n* increases from 7.40 to 33.90%.

Our proposed equation, along with other equations gathered from the literature (Table 5), can be evaluated by comparing the observed values of the response variable ${(y}_{i})$ to the predicted values of the response variable $\left({\hat{y}}_{i}\right)$. Two commonly used measures for this comparison are the root mean square error (RMSE) and the Mean Absolute Deviation (MAD), which are calculated using Equations (3) and (4), respectively. By using both RMSE and MAD, we obtain a more comprehensive understanding of the model’s predictive performance. RMSE highlights the overall fit and the impact of larger prediction errors, while MAD focuses on the average error magnitude, offering insight into the consistency of the model across all data points. These metrics, in conjunction with numerical accuracy, provide a deeper analysis of the model’s reliability and robustness, especially in cases where certain extreme values might disproportionately influence RMSE.

(3) $$RMSE=\sqrt{\frac{\sum {\left(y_{\left(i\right)}-{\hat{y}}_{\left(i\right)}\right)}^{2}}{num}}$$

(4) $$MAD=\frac{\sum \left|y_{\left(i\right)}-{\hat{y}}_{\left(i\right)}\right|}{num}$$

where *num* is the number of observations. It is desirable to have small RMSE and MAD for a good predictability.

Table 5 summarizes ten relationships for predicting the *UCS* of carbonate rocks reported in the literature. According to the independent testing dataset that contains 75 data points from our UC tests on carbonate rocks from Wyoming, along with UC test data from the literature (shown in Table 1 and Table 2), the proposed Equation (2) yields better *UCS* predictions than the other equations according to the lowest RMSE and MAD values as summarized in Table 5. The better performance of the proposed model is attributed to the large dataset of diverse carbonated formations used in the development. The testing dataset from the literature is specific to the rock type for which each equation was developed. For instance, the equation by Farquhar et al. [6] was compared with our proposed equation using the entire testing dataset, as it was developed for carbonate rocks in general. On the other hand, the equation by Hatzor and Palchik [10] was compared with our proposed equation based on the dolomite data of the testing dataset only as the equation of Hatzor and Palchik [10] was developed for dolomite only. The validity of these relationships is assessed based on how accurately they predict the *UCS* for the specific rock types for which they were originally derived. However, uniaxial compression testing is limited by its inability to replicate the lateral confinement present in natural geological settings. The lack of confinement allows unrestricted lateral expansion, often leading to lower measured strength values.

### 3.2. Results of Triaxial Compressive Strength ($\sigma_{1}$)

#### Effect of Porosity and Confining Pressure

**Figure 4:**
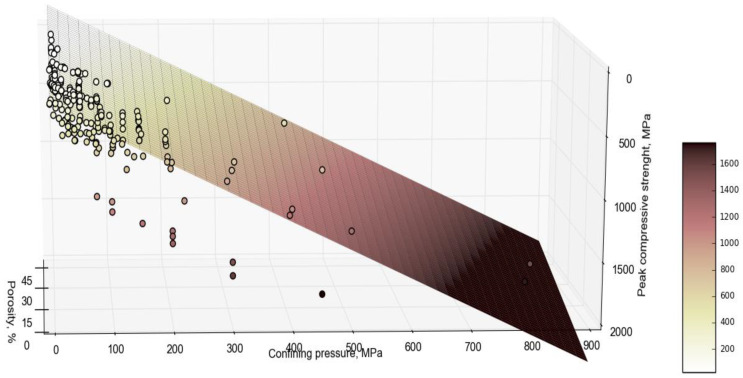
The linear relationship between peak compressive strength, porosity, and confining pressure, highlighting a positive correlation with confining pressure and a negative correlation with porosity.

In this study, the combined effect of *n* and $\sigma_{3}$ is further investigated using experimental data from Wyoming and the literature summarized in Table 3 and Table 4. As shown in Figure 4, the $\sigma_{1}\;$ decreases with the increase in *n* and increases with $\sigma_{3}$. Generally, an increase in *n* leads to a decrease in $\sigma_{1}\;$ due to the higher heterogeneity and the presence of pores, voids, and microcracks in the rock structure, which are considered weak points [74]. In contrast, the mean $\sigma_{1}$ tends to increase with higher $\sigma_{3}\;$ due to the strengthening effect of confinement on compressive strength.

The proposed Equation (5) describes the linear relationship between porosity (*n*) in %, $\sigma_{3}\;$ in MPa, and the true mean $\sigma_{1}$ in MPa based on the training dataset (Table 3 and Table 4), which contains 254 data points (11 are from Wyoming and the remaining ones are from the literature) as shown in Figure 4. While the proposed model provides a good fit within the observed range of data, it may not fully capture any non-linear relationships that might exist at higher or lower porosity values (i.e., extreme values of porosity). Additionally, linear regression assumes a constant rate of change across all levels of porosity, which may not hold in cases where the effect of porosity diminishes or accelerates at the extremes. Therefore, further investigation using non-linear models or transformation of the data may be necessary to better model these extreme behaviors. For samples with similar *n* values, the strength increases as $\sigma_{3}$ increases. For instance, increasing *n* from 1 to 5% at a $\sigma_{3}\;$ of 5 MPa results in a decrease in $\sigma_{1}$ from 255.70 to 221.70 or a 13% strength reduction. In contrast, at a constant *n* of 5%, increasing the $\sigma_{3}\;$ from 5 to 10 MPa increases the $\sigma_{1}\;$ from 221.7 to 233.15 MPa, indicating a 5% increase in the compressive strength.

(5) $$\hat{\sigma_{1}}=252.74-8.499\;n\%+2.29\;\sigma_{3}$$

### 3.3. Young’s Modulus (E)

**Figure 5:**
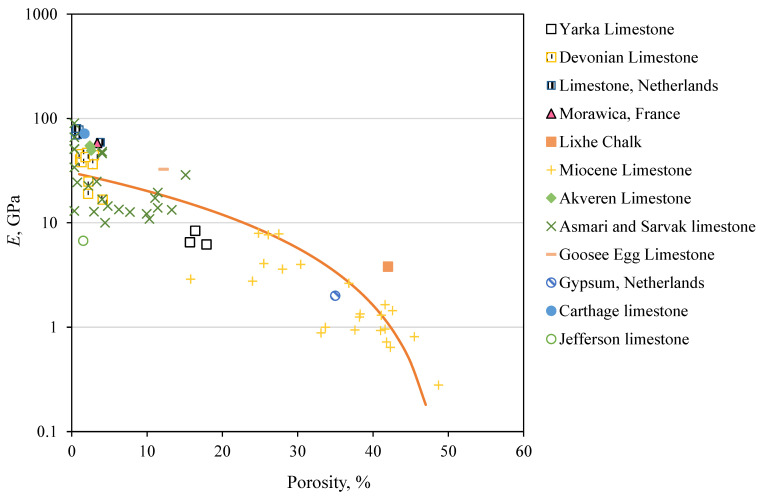
Power relationship showing the decrease in mean Young’s modulus (*E*) as porosity (*n*) increases.

Young’s modulus is a crucial parameter for characterizing rock deformability, as it directly measures the rock stiffness [77]. The estimation of the rock Young’s modulus is essential for designing hydraulic fracturing operations and ensuring the sustainability of geomaterial exploration and extraction. Young’s modulus plays a key role in assessing the deformation characteristics of the rock during the injection of high-pressure fluids. The dynamic testing measures the rock’s response to rapid stress changes, while static testing evaluates the rock’s behavior under long-term, sustained stress. By combining these two approaches, engineers gain a more comprehensive understanding of how a rock will behave under both immediate and long-term loading conditions. This is crucial in predicting the rock’s ability to fracture and the extent of fluid propagation during hydraulic fracturing processes [22]. However, direct measurement is not always feasible in practice [78]. The data presented in Table 1 and Table 2 are randomly split into training and testing datasets. The training dataset includes 79 data points, while the testing dataset contains 66 data points from various formations, including those from Wyoming and the literature. The relationship between *E* and *n* according to the training dataset is plotted in Figure 5. The plot illustrates a power relationship that describes the decrease in mean *E* as *n* increases, with the exponent 1.82 in Equation (6) capturing the sensitivity of *E* to changes in *n*. The fitted relationship indicates that the mean $E_{o}$ at zero porosity is 30.31 GPa. The variability in *E* reflects the geological processes that the rock undergoes such as compaction, precipitation, and dissociation [79].

Equation (6) describes a power decrease in the predicted Young’s modulus ($\hat{E}$) with an increase in the porosity. Past studies related to the predicted *E* to *n* are summarized in Table 6 [6,7,13,14].

(6) $$\hat{E}\left(GPa\right)=30.31\times {\left(1-0.02\;n\right)}^{1.82}$$

The performances of various prediction equations, including our proposed Equation (6) for carbonate rocks are demonstrated in Table 6 in terms of RMSE and MAD. According to the testing dataset, the proposed Equation (6) has the lowest RMSE and MAD values compared to those from the literature, indicating a better prediction of *E*. The enhanced performance of our model can be attributed to its foundation on a more representative and diverse dataset. By incorporating data from various geological formations, including those from Wyoming and other literature sources, our model is better equipped to generalize across different rock types and conditions. This broader dataset enhances the model’s predictive accuracy, giving it a more comprehensive basis compared to models that rely on more limited or less varied data.

## 4. Discussion

### 4.1. Expanded Wing Crack Model

#### 4.1.1. Background

**Figure 6:**
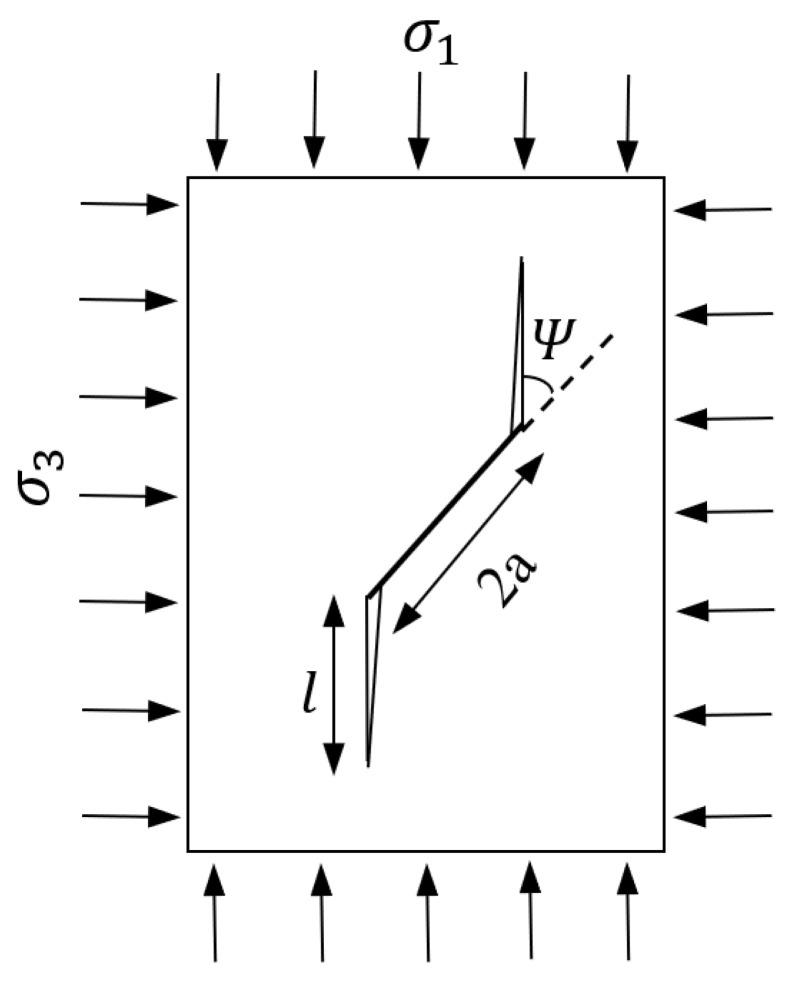
Wing crack growth from an inclined crack under compression (adapted from Ashby and Hallam [17]).

A wing crack model for the growth and interaction of cracks in brittle solids under compression was developed by Ashby and Hallam [18]. Figure 6 illustrates a plate with a pre-existing crack of length 2a, inclined with an angle of Ψ relative to the major principal stress $\sigma_{1}$ direction. Tensile cracks that originate from the tips of pre-existing microcracks and propagate as the stress increases are associated with brittle rock failure.

The expanded wing crack model proposed by the authors [17] states that once wing cracks are initiated, an increase in the applied stress causes further sliding of the main crack, which in turn triggers the growth of wing cracks in parallel to the direction of the maximum applied stress [18]. The stress intensity ($K_{I}$) due to cracks initiation is given by

(7) $$K_{I}=\frac{\sigma_{1}\sqrt{\pi a}}{\sqrt{3}}\times \{(1-\lambda ){\left(1+\mu^{2}\right)}^{1/2}-\left(1+\lambda \right)\;\mu \}$$

where a represents the half-length of the crack, λ is the stress ratio ($\sigma_{3}/\sigma_{1}$), and μ is the coefficient of friction which is defined as tanφ. As the stress increases, these cracks begin to interact further. The governing equation for the stress intensity factor ($K_{I}^{I}$) resulting from microcracks interaction is given by Equation (8)

(8) $$K_{I}^{I}=\frac{\sqrt{2D\times \left(L+\alpha \right)\times \pi a}}{\pi }\;\sigma_{1}\times \sqrt{\left\{\left[1-\frac{8}{\pi }\;D\;\lambda \;{\left(L+\alpha \right)}^{3}\right]\left[1-\frac{2}{\pi }\;D\;\lambda \;{\left(L+\alpha \right)}^{3}\right]\right\}}$$

where $\alpha =cos\Psi =\frac{1}{\sqrt{2}}$, since the angle under the maximum tensile stress is $45^{{}^{\circ}}$, and therefore, α =0.71. D represents the initial level of damage and equals to $\pi a^{2}N_{A}$ where $N_{A}$ denotes the number of initial cracks per unit area.

By combining the stress contributions from external compressive loading, as given by Equation (7), and crack interaction, as described by Equation (8), the total stress intensity (*K_I_*) at the crack tip can be expressed as

(9) $$K_{I}=\frac{\sigma_{1}\sqrt{\pi a}}{\sqrt{3}}\times \{\left(1-\lambda \right){\left(1+\mu^{2}\right)}^{1/2}-\left(1+\lambda \right)\;\mu \}+\frac{\sqrt{2\;D_{o}\times \left(L+\alpha \right)\times \pi a}}{\pi }\;\sigma_{1}\times \sqrt{\left\{\left[1-\frac{8}{\pi }\;D_{o}\;\lambda \;{\left(L+\alpha \right)}^{3}\right]\left[1-\frac{2}{\pi }\;D_{o}\;\lambda \;{\left(L+\alpha \right)}^{3}\right]\right\}}$$

The critical crack length represents the maximum growth length of the crack before failure occurs. Under triaxial compression, crack growth remains stable due to the confinement effect, meaning that the critical crack length is primarily determined by crack interaction. Therefore, the normalized critical crack length (*L_cr_*) can be determined by differentiating the stress intensity ($K_{I}^{I}$) caused by crack interaction, as given by Equation (8), with respect to the crack length *L*, and setting the derivative $\frac{dK_{I}^{I}}{dL}$ equal to zero. The L_cr,_ as given in Equation (10), defines the unstable growth regime of cracks emanating from flaw tips

(10) $$L_{cr}=\sqrt[{3}]{\frac{0.382}{\lambda D}}-0.71$$

By substituting *L* with *L_cr_* given by Equation (10) into Equation (9) and setting $K_{I}=K_{IC}$, and using the value of α=0.71 and the coefficient of friction of carbonate rocks μ=0.60 (corresponding to a mean friction angle of 31⁰ for carbonate rocks [11]), the stress ratio λ (where $\lambda =\sigma_{3}/\sigma_{1}$) can be determined. This, in turn, allows the prediction of the peak compressive strength ($\sigma_{1}$).

(11) $$\frac{k_{Ic}\lambda }{\sigma_{3}\sqrt{\pi a}}=\frac{1}{\sqrt{3}}\left(0.57-1.77\;\lambda \right)+\frac{0.927\sqrt{D}}{\pi }\sqrt[{6}]{\frac{0.382}{\lambda D}}$$

The fracture toughness $k_{Ic}$ is assumed to be 0.2 MPa$\sqrt{m}$ based on fracture measurements performed on calcite [80]. Results of crack initiation of triaxial compression experiments conducted on Solenhofen Limestone by Heard [55] indicated that the initial flaw size (2*a*) is 0.05 mm. The initial damage level, D, was set at 0.15, based on a microcrack model for brittle solids developed by Ashby and Sammis [81]. By substituting these parameters and solving the stress ratio λ by equating both sides of Equation (11), the major principal stress, $\sigma_{1}$, can be determined as a function of the corresponding confining pressure.

#### 4.1.2. Validation of the Expanded Wing Crack Model

**Figure 7:**
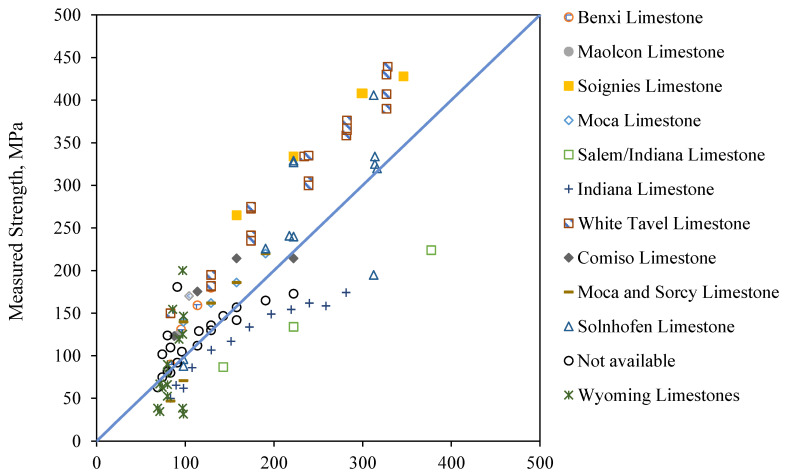
Comparison of measured and predicted compressive strengths for carbonate rocks, showing a good accuracy in predicting the compressive strength.

Figure 7 presents a comparison between the compressive strengths measured from triaxial experiments and the compressive strengths predicted by the expanded wing crack model for various limestone formations. The focus on limestone in this study was based on the availability of robust experimental reference data for both the fracture toughness and flaw size parameters in the wing crack model, specifically for limestone [53,80]. Unfortunately, similar comprehensive experimental data for fracture toughness and flaw size are not available for other rock types at this time. As such, the model’s direct applicability to other rock types remains uncertain without the necessary reference values. The model demonstrates a good level of accuracy in predicting the compressive strength, with a mean bias (i.e., the ratio of the measured to the predicted strength) of 1.07. However, some scatterness around the 1:1 line is observed, and this is related to the inherent variability in rock properties, influenced by factors such as mineral composition and microstructural heterogeneities. Scale effects also play a role, as laboratory relationships may not fully capture the strength variations observed in larger rock masses.

Although regression models are valuable predictive tools, they exhibit inherent limitations, especially when applied to real-world scenarios. A primary constraint is that these models typically assume a linear relationship between variables, which may not accurately reflect the complexities of non-linear field conditions. Furthermore, the accuracy of regression models is highly dependent on the quality and representativeness of the training data; any biases or errors in the dataset can result in significant inaccuracies in field predictions.

The relationship between mechanical properties (e.g., *UCS*, Young’s modulus) and porosity may be altered by factors such as weathering, mineral alteration, or fracture development over time. In some materials, higher porosity might not necessarily correlate with lower strength if other factors (such as cementation or consolidation) are at play, complicating the use of standard models. These factors can introduce significant variability in mechanical behavior, making it challenging to generalize predictions across different environments. Given the complexity and variability of these processes, further research is needed to better understand the interactions between porosity and mechanical properties, particularly in field conditions.

## 5. Conclusions

To better understand the mechanical behavior of carbonate rocks and to formulate new predictive equations, uniaxial and triaxial compression tests were conducted on three limestones, two granites, and one dolostone rock. Additionally, data from previous studies on carbonate rocks were collected from the published literature. The findings highlight the significant effect of porosity (*n*) on the uniaxial compressive strength (*UCS*) and Young’s modulus (*E*) of carbonate rocks. Furthermore, the combined influence of porosity (*n*) and confining pressure ($\sigma_{3}$) on the triaxial compressive strength ($\sigma_{1}$) was also examined.

The regression analysis results revealed that both *UCS* and *E* were related to the porosity (*n*) by a power relationship, while the triaxial $\sigma_{1}$ was related linearly with the *n* and $\sigma_{3}$. The accuracy of the proposed equations in predicting strength and stiffness was assessed by comparing them with other equations of previous studies in the literature, testing datasets for validation. Our proposed equations demonstrated better predictability for both the strength and Young’s modulus than other equations, as evidenced by lower RMSE and MAD values.

Additionally, an expanded wing crack model was applied to limestone to predict the triaxial compressive strength ($\sigma_{1}$) by considering parameters such as the initial flaw size, fracture toughness of the rock, the coefficient of friction, the initial level of damage, and the confining pressure. The proposed model effectively predicted the compressive strength, demonstrating a strong agreement between the measured and predicted values. Further research may explore the application of these models to other rock types and environmental conditions to further refine their applicability.

The findings from this study have important implications for real-world applications, particularly in petroleum engineering and geomechanical modeling. The enhanced predictive models can support more accurate subsurface evaluations, including reservoir characterization, hydraulic fracturing, and wellbore stability assessments. By improving the prediction of rock behavior under varying conditions, these models can aid in optimizing drilling and production strategies, minimizing risks, and enhancing the overall efficiency and safety of subsurface operations.

## Figures and Tables

**Table 1:** Summary of the UC test results of carbonate rock formations in Wyoming.

Rock Type	Formation	Geological Age	*n*, %	*UCS*, MPa	*E*, GPa
Limestone	Goose Egg	Permian	12.41	61.27	29.61
Limestone	Jefferson	Devonian	2.12	11.33	15.37
Granite	NA	Precambrian	0.81	87.97	34.41
Granite	Sherman	Proterozoic	3.49	26.77	17.01
Dolostone	Big Horn	Ordovician	8.50	23.13	9.83

*n*—Porosity in percentage; *UCS*—uniaxial compressive strength in MPa; *E*—Young’s modulus in GPa; and NA—not available.

**Table 4:** Summary of triaxial compression test results of carbonate rocks collected from literature.

Formation, (Location)	Country of Origin	*n*, %	$\sigma_{3}$, MPa	$\sigma_{1}$, MPa	Reference
Soignies Limestone	Belgium	0.40	2–90	206.00–443.00	[43]
Soignies Limestone	Mons-Belgium	0.40	30–90	265.00–428.00	[42]
Moca Limestone		8.00	10–40	140.00–220.00	
Sorcy Limestone		29.50	5–10	47.00–71.00	
Saint Maximin Limestone	Italy, France	37.00	3–6	20.00–25.00	[44]
Salem/Indiana Limestone	United States	16.90	25–400	87.00–544.00	[45]
Tavel Limestone	United States	10.40	10–50	221.00–313.00	[46]
Indiana Limestone	United States, Italy	13.40	5–10	45.00–62.00	[46,47]
Indiana Limestone	United States	19.40	7–69	65.50–174.40	[48]
White Tavel Limestone	France	14.70	20–85	181.33–430.00	[49,50]
Comiso Limestone	Italy	10.10	7–30	123.23–214.41	[51]
Solnhofen Limestone	Germany	3.00	10–50	336.00–478.00	[52]
Solnhofen Limestone	United States	4.80	17–81	277.00–491.00	[53]
Solnhofen Limestone	Italy	5.90	100–800	530.00–1730.00	[54]
Solnhofen Limestone	Germany	1.70	20–500	493.00–1264.00	[55]
Solnhofen Limestone	United States	4.80	0.1–98	270.76–490.33	[53]
Intact Solnhofen Limestone	NA	3.70–5.50	6–195	311.00–703.00	[56]
Oak Hall Limestone	United States	0.30	18–220	388.00–1000.00	[53]
Indiana Limestone	Canada	0.15	2–50	75.00–173.00	[34]
Benxi Limestone	China	NA	5–20	90–180	[57]
Maokou Limestone	China	0.09	4–12	80.90–170.00	[58]
Reef Limestone	South China Sea	0.02	1–8	63.00–181.00	[35]
Xuzhou Limestone	NA	NA	5–30	110.00–157.00	[59]
Comiso Limestone	Italy	0.10	7–50	123.23–214.41	[51]
Karst Limestone	China	NA	5–25	131.00–288.00	[60]
Majella Grainstone	Italy, France	30.00	5–21	32–46	[44]
Georgia Marble	United States	2.70	7–69	83.00–228.00	[48]
Carrara Marble	Italy	1.10	50–800	270.00–1530.00	[61]
Carrara Marble	NA	1.10	1.72–34.5	80.31–247.99	[62]
Carrara Marble	Italy	1.10	5–450	100.00–770.00	[61]
Wombeyan Marble	Australia	0.90	0.1–98	69.73–332.45	[63]
Blair Dolomite	Germany	0.90	50–450	549.00–1760.00	[64]
Cold-pressed Aragonite	Germany	10.60–22.30	10–195	84.00–661.00	[65]
Cold-pressed Calcite		6.90–16.20	10–150	89.00–500.00	
Cold-pressed Solnhofen Limestone		7.40–15.80	10–150	96.00–485.00	
Gypsum	Italy	0.50	2–95	19.80–83.00	[66]

*n*—Porosity (%); $\sigma_{3}$—confining pressure (MPa); $\sigma_{1}$—compressive Strength (MPa); and NA—not available.

**Table 5:** Assessment of prediction equations for *UCS* based on testing dataset.

Rock Type	Equation from Literature	Equation from Literature		Reference	Testing Dataset Size	Our Proposed Equation (2)	
		RMSE	MAD			RMSE	MAD
Carbonate rocks	$UCS=174.8\;e^{-9.3\;n}$	48.64	37.15	[6]	76	29.29	21.8
Carbonate rocks, *n* < 0.3	$UCS=-28.56\ln\left(n\right)+105.05$	23.79	17.64	[8]	60	31.16	25.72
Dolomite	$UCS=\frac{\pi \;{\times E}^{0.25}}{{n\%}^{0.45}\times \sqrt{d_{m}}}$	51.51	49.08	[10]	8	27.96	22.49
Gypsum (2 data pts)	$UCS=16.68\times e^{-0.8193\;w\%}$	33.69	33.68	[75]	2	4.24	3.04
Limestone	$UCS=13.8\times E^{0.51}$	24.3	28.79	[76]	64	24.3	27.02
Dolomite	$UCS=25.1\times E^{0.34}$	23.92	19.21		8	27.96	22.49
Limestone and Dolomite	$UCS=276\times {\left(1-3\times n\right)}^{2}$	103.84	69.73	[5]	72	22.33	13.7
Carbonate rocks with 0.05 < *n* < 0.2 and 30 < *UCS* < 150	$UCS=143.8\times e^{-6.95\;n}$	46.9	40.03		57	33.1	26.77
Carbonate rocks with 0.05 < *n*< 0.2 and 30 < *UCS* < 150	$UCS=135.9\times e^{-4.8\;n}$	44.95	51.12		57	26.77	33.1
Carbonate rocks	$UCS=-7.7\ln\left(n\right)+74.5$	32.71	26.07	[7]	76	26.39	17.48

*UCS*—Uniaxial compressive strength in MPa; *w*—water content in percentage; *n*—rock porosity in percentage; RMSE—root mean square error; and MAD—Mean Absolute Deviation.

**Table 6:** Assessment of prediction equations based on testing dataset.

Rock Type	Equation	Reference	Testing Dataset Size	Equation from Literature		Our Proposed Equation (6)	
				RMSE	MAD	RMSE	MAD
Carbonate rocks	$E=69.05\;e^{-6\;n}$	[7]	66	20.02	14.87	15.8	10.72
Carbonate rocks, *n* < 0.3	$E=86.094\;e^{-5.34\;n}$	[13]	46	35.94	31.95	18.89	14.89
Carbonate rocks, *n* < 0.3	$E={\left(\frac{UCS}{2.94}\times n^{0.088}\right)}^{1.2}$	[14]	46	13.49	19.47	10.72	15.8
Carbonate rocks	$E=36.6\;{\left(0.91\right)}^{n\%}$	[6]	66	15.21	10.35	15.8	10.71

RMSE—Root mean square error; MAD—Mean Absolute Deviation; *n*—porosity in percentage; and *E*—Young’s modulus in GPa.

## Data Availability

The raw data supporting the conclusions of this article will be made available by the authors on request.
